# Specific treatment of problems of the spine (STOPS): design of a randomised controlled trial comparing specific physiotherapy versus advice for people with subacute low back disorders

**DOI:** 10.1186/1471-2474-12-104

**Published:** 2011-05-20

**Authors:** Andrew J Hahne, Jon J Ford, Luke D Surkitt, Matthew C Richards, Alexander YP Chan, Sarah L Thompson, Rana S Hinman, Nicholas F Taylor

**Affiliations:** 1 Musculoskeletal Research Centre and School of Physiotherapy, La Trobe University, Bundoora, Victoria 3086, Australia; 2 Spinal Management Clinics of Victoria, LifeCare Health, Victoria, Australia; 3 Department of Physiotherapy, The University of Melbourne, Australia

## Abstract

**Background:**

Low back disorders are a common and costly cause of pain and activity limitation in adults. Few treatment options have demonstrated clinically meaningful benefits apart from advice which is recommended in all international guidelines. Clinical heterogeneity of participants in clinical trials is hypothesised as reducing the likelihood of demonstrating treatment effects, and sampling of more homogenous subgroups is recommended. We propose five subgroups that allow the delivery of specific physiotherapy treatment targeting the pathoanatomical, neurophysiological and psychosocial components of low back disorders. The aim of this article is to describe the methodology of a randomised controlled trial comparing specific physiotherapy treatment to advice for people classified into five subacute low back disorder subgroups.

**Methods/Design:**

A multi-centre parallel group randomised controlled trial is proposed. A minimum of 250 participants with subacute (6 weeks to 6 months) low back pain and/or referred leg pain will be classified into one of five subgroups and then randomly allocated to receive either physiotherapy advice (2 sessions over 10 weeks) or specific physiotherapy treatment (10 sessions over 10 weeks) tailored according to the subgroup of the participant. Outcomes will be assessed at 5 weeks, 10 weeks, 6 months and 12 months following randomisation. Primary outcomes will be activity limitation measured with a modified Oswestry Disability Index as well as leg and back pain intensity measured on separate 0-10 Numerical Rating Scales. Secondary outcomes will include a 7-point global rating of change scale, satisfaction with physiotherapy treatment, satisfaction with treatment results, the Sciatica Frequency and Bothersomeness Scale, quality of life (EuroQol-5D), interference with work, and psychosocial risk factors (Orebro Musculoskeletal Pain Questionnaire). Adverse events and co-interventions will also be measured. Data will be analysed according to intention to treat principles, using linear mixed models for continuous outcomes, Mann Whitney U tests for ordinal outcomes, and Chi-square, risk ratios and risk differences for dichotomous outcomes.

**Discussion:**

This trial will determine the difference in outcomes between specific physiotherapy treatment tailored to each of the five subgroups versus advice which is recommended in guidelines as a suitable treatment for most people with a low back disorder.

**Trial registration:**

Australia and New Zealand Clinical Trials Register (ANZCTR): ACTRN12609000834257.

## Background

Low back disorders (LBD) affect up to 84% of people at some point in their lives [1], creating high rates of activity limitation, work absence, impaired quality of life and the need for medical care [2]. The economic burden resulting from LBD is high [3]. While it has often been thought that the prognosis for most people with acute LBD is favourable, ongoing or recurring pain and activity limitation are common [4-7].

A variety of treatments have been developed and evaluated for people with LBD. While several have been shown to be superior to placebo or no treatment, comparisons between treatments rarely demonstrate clinically meaningful differences [8-10]. One proposed explanation for these results is clinical heterogeneity of participants within randomised controlled trials [11,12]. Sample heterogeneity can diminish the chance of finding a significant treatment effect due to the reduced proportion of the sample for whom the treatment is appropriate [12].

In an attempt to overcome participant heterogeneity, a number of classification systems have been proposed for identifying subgroups of people with LBD who might respond more predictably to specific treatment [12,13]. It has been recommended that future randomised controlled trials should incorporate the use of subgroups in the hope that larger effect sizes may result [12,14]. This approach is consistent with clinical practice where most clinicians aim to identify subgroups and provide a tailored treatment program [11].

One approach to classification of LBD is to subgroup people based on the known or hypothesised causal factors [12,15]. This allows specific treatments to be developed targeting the causal mechanisms of the disorder [12,16]. The evolution of surgical discectomy to relieve radiculopathy cased by a herniated intervertebral disc is an example of this method targeting a pathoanatomical causal mechanism [17,18]. In that case, validation of the classification approach is evident from studies showing that discectomy surgery performed on participants with radiculopathy from a herniated disc results in superior short and intermediate term pain and activity outcomes compared to other treatments [19,20].

Given the multifactorial nature of LBD, factors other than the pathoanatomical source of pain also need to be considered in a robust classification approach [12], including neurophysiological [21,22] and psychosocial components of LBD [23,24]. The duration of injury is also considered an important factor in the presentation and prognosis of people with LBD [24-26]. We are interested in investigating specific physiotherapy treatment strategies in the subacute population of LBD, where spontaneous recovery is less rapid and the complex issues associated with chronic pain are less likely to be fully entrenched [4,6,27]. In the context of such a population, we have developed specific treatment protocols for five LBD subgroups that consider pathoanatomical, neurophysiological and psychosocial mechanisms. The five subgroups are i) lumbar disc herniation with associated radiculopathy (Ford JJ, Hahne AJ, Chan AYP: A classification and treatment protocol for low back disorders: Part 3- Functional restoration for intervertebral disc related problems, submitted); ii) reducible discogenic pain (Ford JJ, Surkitt LD, Hahne AJ: A classification and treatment protocol for low back disorders: Part 2- Directional preference management for reducible discogenic pain, submitted); iii) non-reducible discogenic pain (Ford JJ, Hahne AJ, Chan AYP: A classification and treatment protocol for low back disorders: Part 3- Functional restoration for intervertebral disc related problems, submitted); iv) zygapophyseal joint dysfunction [28] and; v) multi-factorial persistent pain (Ford JJ, Richards MC, Hahne AJ: A classification and treatment protocol for low back disorders: Part 4- Functional restoration for multi-factorial persistent pain, submitted). These five subgroups have been chosen based on i) their common recognition by clinicians [29-31]; ii) their description in another pathoanatomical classification system [15,32] and; iii) significant evidence of subgroup reliability and validity [15,32,33].

A randomised controlled trial is planned to evaluate the effectiveness of the subgroup-specific treatment. Advice will be the comparison intervention as it is recommended in all international guidelines for the management of LBD [34] and has demonstrated efficacy in randomised controlled trials [35-37].

The aim of this paper is to describe the design of a randomised controlled trial comparing specific physiotherapy treatment to physiotherapy advice for people classified into five subacute LBD subgroups.

## Methods/Design

### Study design

**Figure 1:**
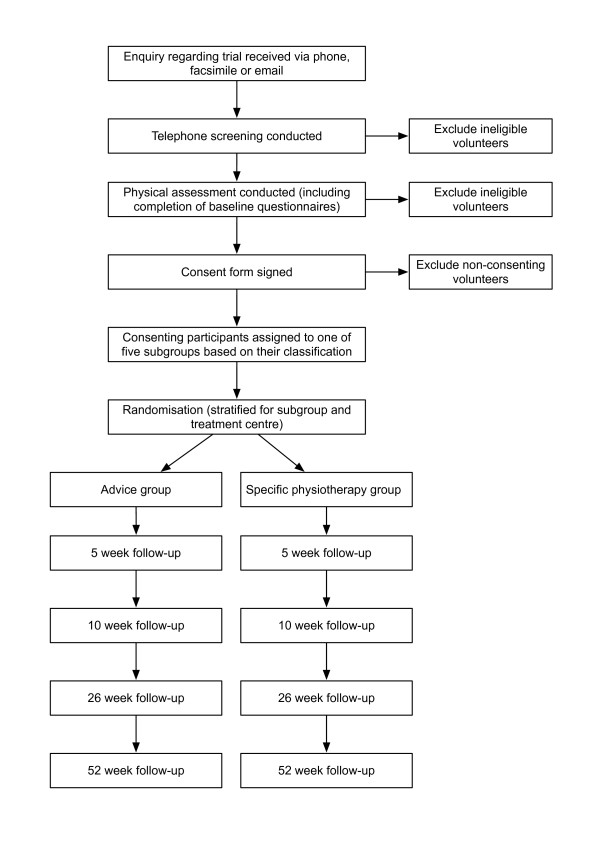
Overview of participant flow through the trial

This will be a multi-centre parallel group randomised controlled trial. An overview of the process of the trial is presented in Figure 1.

### Ethics and registration

The trial has received ethical approval from the La Trobe University Human Ethics Committee, and has been registered with the Australian New Zealand Clinical Trials Registry (#12609000834257).

### Setting

The treatments will be conducted at private physiotherapy practices that are part of the Spinal Management Clinics of Victoria network throughout metropolitan Melbourne and Geelong, Australia. We have chosen this network as a source of treatment centres and practitioners due to existing quality assurance procedures as well as training and mentoring programs that are accessible for the purposes of the trial.

### Eligibility and screening

Participants will be sought via newspaper advertising, public notices, and via referral from medical practitioners and physiotherapists. Potential participants will initially undergo a preliminary screening for eligibility via telephone. These initial selection criteria are common to all subgroups and are outlined in Table 1. Those found to be potentially eligible will be invited to attend a physical examination with one of the treating physiotherapists. The physical examination will i) confirm eligibility; ii) determine which subgroup (if any) the participant fits and; iii) provide descriptive information on the baseline characteristics of participants. The physical examination will involve:

**Table 1:** Eligibility criteria common to all subgroups

Inclusion criteria
1. A primary complaint of either: a. low back pain, defined as pain between the inferior costal margin and the inferior gluteal fold with or without referral into the leg(s) [138,139], or b. referred leg pain, defined as predominately unilateral posterior leg pain extending below the knee, or anterior thigh pain, with or without back pain (disc herniation with associated radiculopathy subgroup only) [56]
2. Duration of current episode of primary complaint lasting between 6 weeks and 6 months (subacute stage of injury [27,140]), with at least a 4 week pain-free period separating the current episode from any previous episodes [141,142]
3. Aged between 18 and 65 (inclusive)
4. Fluency in English sufficient to complete questionnaires and to enable understanding of the intervention
5. Classified into one of the five subgroups of low back disorders being targeted in the trial
6. Agreeing to refrain from other interventions wherever possible for the 10-week treatment period of the trial, aside from consultations with medical practitioners, medication, and any exercises already being undertaken

**Exclusion criteria**

1. An active compensation claim for their back injury, due to the negative influence that this can have on prognosis [125]
2. Active cancer under current treatment, as the treatment of the cancer may interfere with their ability to participate in the trial
3. Signs of cauda equina syndrome based on bladder or bowel disturbance and/or imaging [143]
4. Current pregnancy, or childbirth within the last 6 months, as this could impair ability to undertake exercises, and could also cause back and leg symptoms that are not related to the subgroups under investigation
5. Spinal injections within the last 6 weeks, as we wish to study treatment effects independent to the effects of injections [144]
5. Any history of lumbar spine surgery, as there is already considerable research evaluating the efficacy of post-surgical rehabilitation programs [145]
6. A pain intensity score of less than 2/10 on a 0-10 numerical rating scale due to low severity
7. Minimal activity limitation, evidenced by a baseline ability to walk, sit and stand for one hour or more and no sleep disturbance at night, as we wish to exclude people with low severity
8. Already received more than 5 sessions of physiotherapy with any of the treating physiotherapists prior to enrolment, as these therapists are likely to use many components of the trial treatment protocol on their usual client caseload
9. Inability to walk safely, such as severe foot drop causing regular tripping, as the interventions in the trial include walking for most participants
10. Planned absence of more than one week during the treatment period (such as overseas holidays)

• Observation of the spine for evidence of postural deformity such as a lateral shift or an increased or decreased lumbar lordosis [38], using protocols with acceptable reliability [39,40].

• Measurement of lumbar spine active movements into flexion, extension and lateral-flexion using finger-to-floor measurement methods that have been shown to be reliable [41].

• Lower limb neurological examination in a seated position, which will involve testing of knee jerk and ankle jerk reflexes, myotomal strength testing and dermatomal sensation in response to light touch with a tissue [42]. Acceptable reliability for these tests has been demonstrated in people with suspected lower limb nerve root compression [42].

• Straight leg raise and crossed straight leg raise, which will be considered positive if the participant's usual lower limb symptoms are reproduced at any angle during passive raising of either leg by the examiner [42]. The reliability of this test is considered good when performed on people with suspected nerve root compression [42].

• Prone knee flexion test, which will be considered positive if the participant's usual anterior thigh symptoms are reproduced at any angle [43,44]. The reliability of this test has been shown to be good in people with suspected nerve root compression [42].

• Lumbar spine palpation performed with the participant prone, with the examiner applying pressure centrally over the lumbar spinous processes and unilaterally over the lumbar zygapophyseal joints and/or transverse processes [45]. Where a localised painful or stiff segment is identified, a "mini-treatment" will be undertaken consisting of a 30-second low grade mobilisation of the joint, and the participant's response will be recorded in terms of any changes in pain or range of motion upon repeat testing [28,45]. Studies have demonstrated good reliability for lumbar spine palpation [46,47].

• Mechanical loading strategies including sustained prone positioning and repeated extension movements in a prone position (with or without lateral shift of the pelvis), which will be assessed to determine whether a directional preference is present. Directional preference will be defined as the direction of movements or postures that result in either centralisation of symptoms, sustained decrease in symptoms (by at least 1 point on a 0-10 numerical rating scale) or improvement in range-of-motion following the assessment of mechanical loading strategies [48-50]. Assessing for a directional preference has been shown to be reliable [50,51].

• Determination of the participant's ability to activate the transversus abdominis via a localised inward movement of the lower abdominal wall in a standing position which will be visualised and palpated using a reliable method [52,53]. Each participant's ability to activate the lumbar multifidus via localised generation of tension will also be assessed via palpation of the participant in prone. Although therapist palpation is one recommended method of assessing the activation of multifidus [53], studies evaluating the reliability of this method could not be located.

Participants will also complete the Subjective Complaints Questionnaire, which is a valid and reliable self-administered tool for gaining subjective information relating to the history of back and leg symptoms, the mechanism of onset, and the nature and behaviour of symptoms [54].

### Classification

Data from the Subjective Complaints Questionnaire and the physical examination will be used to classify participants into one of the five pre-defined subgroups, i) disc herniation with associated radiculopathy; ii) reducible discogenic pain; iii) non-reducible discogenic pain; iv) zygapophyseal joint dysfunction and; v) multi-factorial persistent pain. A Microsoft Excel^a ^spreadsheet containing a decision rule algorithm will be used to reliably identify subgroup membership after examination and questionnaire data have been entered.

**Figure 2:**
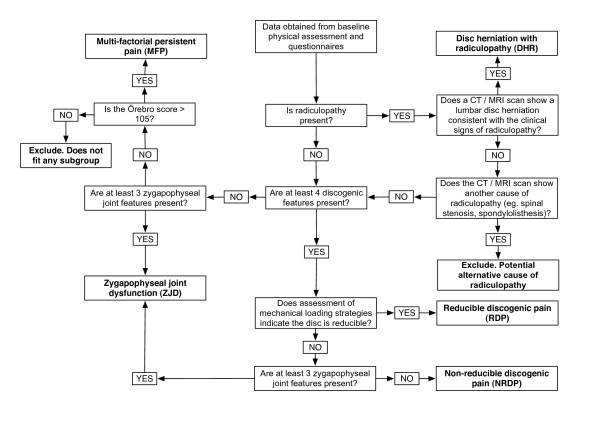
Decision rule algorithm for classifying participants into subgroups

The five subgroups targeted in this trial do not represent an exhaustive classification system for LBD. People who do not fit into one of the subgroups will be excluded from the trial. Detailed justification for each of the subgroups is reported elsewhere (Ford JJ, Surkitt LD, Hahne AJ: A classification and treatment protocol for low back disorders: Part 2- Directional preference management for reducible discogenic pain, submitted; Ford JJ, Hahne AJ, Chan AYP: A classification and treatment protocol for low back disorders: Part 3- Functional restoration for intervertebral disc related problems, submitted; Ford JJ, Richards MC, Hahne AJ: A classification and treatment protocol for low back disorders: Part 4- Functional restoration for multi-factorial persistent pain, submitted; [15,28,32]). An overview of the decision rule algorithm for classifying participants into subgroups is presented in Figure 2. A description of the five subgroups to be included in the trial is presented below.

#### Disc herniation with associated radiculopathy

To be classified in this subgroup, participants will have i) referred leg symptoms (below the knee for L3/4, L4/5 or L5/S1 herniations, or into the anterior thigh for L1/2, L2/3 or L3/4 herniations); ii) at least one clinical examination sign suggestive of radiculopathy (reduced reflex, reduced dermatomal sensation, reduced myotomal strength, reproduction of usual leg symptoms on straight leg raise or reproduction of usual anterior thigh symptoms on prone-knee-flexion testing) and; iii) a Computerised Tomography (CT) or Magnetic Resonance Imaging (MRI) scan demonstrating a lumbar disc herniation consistent with the examination findings [55,56].

#### Reducible discogenic pain

To be classified in this subgroup, participants will present with at least four out of nine features indicative of discogenic low back pain. The features have been identified through literature searching, consideration of causal mechanisms and the results of a Delphi study of experts in the field (Chan AYP, Ford JJ, McMeeken JM, Wilde VE: Features of non-reducible discogenic low back pain: survey of an international expert panel with the Delphi technique, submitted). The features are:

1) Presence of low back pain with or without leg pain

2) Sitting limited to less than 60 minutes

3) Symptoms worse the next morning or next day following the initial injury

4) History of working in a job involving manual handling

5) A mechanism of injury associated with flexion/rotation and/or compression loading

6) At least some difficulty with forward bending

7) At least some difficulty with lifting

8) At least some difficulty with sit-to-stand

9) At least some difficulty with coughing/sneezing.

Participants will also demonstrate a directional preference in response to mechanical loading strategies on physical examination. The presence of a directional preference has been proposed as identifying people likely to have discogenic pain where a posterior or posterolaterally migrated nucleus pulposus can be "reduced" into a more central and non pain provoking position [15,50].

#### Non-reducible discogenic pain

To be classified in this subgroup, participants will have at least four out of nine signs of discogenic pain as outlined above for the reducible discogenic pain subgroup. However, participants who do *not *demonstrate a directional preference in response to mechanical loading strategies will be classified in the non-reducible (rather than reducible) discogenic pain subgroup provided they do not satisfy the selection criteria for the zygapophyseal joint dysfunction group.

#### Zygapophyseal joint dysfunction

This group will comprise participants believed to have zygapophyseal joint dysfunction as the primary source of their back symptoms. To be classified in this subgroup, participants will have at least three of the following four features of zygapophyseal dysfunction, i) presence of unilateral low back pain; ii) a regular compression pattern (pain reproduced with lumbar extension and ipsilateral lateral-flexion movements); iii) localised pain on ipsilateral passive postero-anterior accessory movement applied through the transverse process or the zygapophyseal joint at one or two segments and; iv) improvement in pain or range-of-movement following a "mini-treatment" of manual therapy directed at the zygapophyseal joint [28].

#### Multi-factorial persistent pain

Participants without a clear pathoanatomical classification (ie. they do not fit one of the other four pathoanatomical subgroups), who also have an Orebro Musculoskeletal Pain Questionnaire score greater than 105, will be classified as belonging to this subgroup. In these participants it is hypothesised that psychosocial and/or neurophysiological factors may be negatively impacting their recovery.

### Randomisation and allocation

Eligible participants who provide written consent to participate will be randomised into one of two treatment groups; advice or specific physiotherapy treatment. A randomisation schedule will be prepared in advance by a researcher (NT) who will have no contact with any participants throughout the trial and will not be involved in the recruitment, screening, assessment, enrolment or treatment process. The randomisation sequence will be generated using a web-based randomisation program (http://www.randomization.com) with the sequence transferred into a computer spreadsheet. Permuted block randomisation with random block lengths will be used to avoid unequal group sizes [57]. Randomisation will be stratified for subgroup and treatment centre by generating a separate schedule for each combination of subgroup and centre.

Allocation of participants in accordance with the randomisation schedule will be undertaken by an administrative assistant who will be the only person with access to the allocation spreadsheet during the trial. The administrative assistant will not be involved in the recruitment, screening, assessment, enrolment, or treatment of participants, and will be located at a separate site to all treatment centres [58]. To enrol a participant, the treating physiotherapist will email the consenting participant's name and date of birth to the administrative assistant along with their subgroup classification and treatment centre. These details will be entered into the allocation spreadsheet and the next treatment allocation and participant identification number will be emailed back to the treating physiotherapist.

### Treatment protocols

Two different 10-week physiotherapy programs will be compared. The protocols for each intervention will be outlined in a detailed treatment manual, supplemented by electronic "clinical notes" which will contain an outline of the treatment protocol on a session-by-session basis. The clinical notes will prompt treating physiotherapists regarding the mandatory and optional treatment components for each session. The clinical notes will require the treating physiotherapists to document assessment/reassessment findings, clinical reasoning/decision making rationale, treatment provided and response to treatment during each session.

#### Comparison intervention: physiotherapy advice

Participants allocated to the physiotherapy advice intervention will attend two 30-minute physiotherapy sessions with one of the treating physiotherapists during a 10-week period. The intervention will follow the approach described by Indahl et al. [35], which consists of providing a pathological explanation of the participant's pain, reassurance regarding the generally favourable prognosis of their condition, advice to remain active and instruction regarding correct lifting technique. Advice is recommended in all international low back pain guidelines [34] and has demonstrated efficacy in randomised controlled trials [35-37].

#### Primary intervention: specific physiotherapy treatment

Participants allocated to the specific physiotherapy intervention will attend ten 30-minute physiotherapy sessions over a 10-week period. The content of the sessions will relate to known or hypothesised mechanisms underpinning each of the five subgroups. Within each subgroup, treatment methods will be clearly defined and standardised via detailed session-by-session clinical notes that contain a series of decision making algorithms. The algorithms and clinical notes will ensure that essential elements of the treatment program are consistently applied by all physiotherapists across all participants, while still allowing some opportunity for the treatment to be tailored to individual participants. Information provided to participants will be standardised via a series of pre-prepared participant information sheets specific for each subgroup. The treatment components, participant handouts and decision making algorithms are based on existing protocols developed by a senior musculoskeletal physiotherapist (JF). These have been refined via systematic reviews of the effectiveness of interventions for each subgroup that were undertaken by members of the research team, one of which has been published to date [20]. A full day meeting was also attended by five members of the research team and eight physiotherapists who planned to treat participants in the trial, with the aim of evaluating the suitability and clinical utility of the selected treatment components for each subgroup. Separate articles fully describe the rationale and detailed application of the specific physiotherapy treatment protocols for each subgroup (Ford JJ, Surkitt LD, Hahne AJ: A classification and treatment protocol for low back disorders: Part 2- Directional preference management for reducible discogenic pain, submitted; Ford JJ, Hahne AJ, Chan AYP: A classification and treatment protocol for low back disorders: Part 3- Functional restoration for intervertebral disc related problems, submitted; Ford JJ, Richards MC, Hahne AJ: A classification and treatment protocol for low back disorders: Part 4- Functional restoration for multi-factorial persistent pain, submitted; [28]). In the current paper, a summary of the mandatory and optional treatment components for the specific physiotherapy treatment arm is presented below for each subgroup and a list of all components is contained in Table 2.

**Table 2:** Components of each treatment protocol used in the trial

Treatment component	DHR	NRDP	RDP	ZJD	MFP	Advice
Patho-anatomical/physiological explanation including generally favourable prognosis	✔	✔	✔	✔	X	✔
Advice in accordance with Indahl et al. [35]	X	X	X	X	X	✔
Explanation of pain physiology and central sensitisation for ongoing pain with multiple biopsychosocial contributing factors	O	O	O	O	✔	X
Discussion of treatment options available	✔	✔	✔	✔	✔	X
Discussion of timeframes and expectations	✔	✔	✔	✔	✔	X
Posture education including lifting technique	✔	✔	✔	O	X	X
Teaching pacing and graded exposure strategies	✔	✔	✔	O	✔	X
Goal setting (establishment and regular reviews)	✔	✔	✔	✔	✔	X
Specific motor control training (transversus abdominis, lumbar multifidus and pelvic floor)	✔	✔	✔	✔	O	X
Teaching and supervision of functional restoration exercises in the clinic with additional sessions at home	✔	✔	X	X	✔	X
Demonstration of functional restoration exercises for implementation at home	X	X	✔	✔	X	X
Education regarding pain management strategies (pharmacological)	O	O	O	O	O	X
Education regarding pain management strategies (non-pharmacological)	O	O	O	O	O	X
Strategies to control inflammation	O	O	O	O	O	X
Application of strapping tape to lumbar spine	✔	✔	✔	O	X	X
Discussion of strategies to manage work issues	O	O	O	O	O	X
Directional preference management (McKenzie program)...includes mechanical loading strategies, repeated movements, walking program, taping, and postural advice	O	O	✔	X	X	X
Manual therapy	X	X	X	✔	X	X
Relaxation strategies	O	O	O	O	O	X
Sleep strategies	O	O	O	O	O	X
Management of increases in pain	O	O	O	O	X	X
Explanation of improvement in function V's improvement in pain	O	O	O	O	X	X
Cognitive restructuring of counterproductive beliefs (via use of information sheets relating to the above treatment components)	✔	✔	✔	✔	✔	X
Behavioural strategies to support and reinforce the education and information provided and to modify unproductive behaviours	✔	✔	✔	✔	✔	X
Transfer to MFP protocol if inadequate progress with pathoanatomical approach after five sessions	O	O	O	O	X	X
Targeted cognitive restructuring and behavioural modification based on review of the Orebro Musculoskeletal Pain Questionnaire subscales	X	X	X	X	✔	X
Specific discussion of psychosocial barriers as an explanation for failure to recover	O	O	O	O	✔	X
Discharge planning for long-term management	✔	✔	✔	✔	✔	X

✔ = component mandatory, O = component optional/if required, X = component not allowed, DHR = disc herniation with associated radiculopathy, NRDP = non-reducible discogenic pain, RDP = reducible discogenic pain, ZJD = zygapophyseal joint dysfunction, MFP = multi-factorial persistent pain

The main component of treatment in the disc herniation with associated radiculopathy subgroup and the non-reducible discogenic pain subgroup will be a functional restoration program modified for the presence of discogenic pathology. The content of the program will be similar for both subgroups but the nature of the pathoanatomical information will be specific to each subgroup. Specific motor control training targeting the core stabilising muscles (transversus abdominis, lumbar multifidus and pelvic floor muscles) will initially be taught in non-weight bearing positions [53]. This will then be progressed into functional exercises relevant to each participant's work and daily activities with the aim of restoring function and normal motor control during these tasks. A detailed case study of a person with disc herniation with associated radiculopathy who was treated using this approach has been published previously [59].

The main component of the specific physiotherapy treatment for the reducible discogenic pain subgroup will involve directional preference management based on the McKenzie method [38]. Initial and subsequent assessments will identify the direction of repeated movements or sustained positions that lead to improvement or centralisation of the participant's pain. Advice, postural strategies and exercises will then be implemented in order to promote movements and positions corresponding with this directional preference. Once the directional preference management has been established, all participants in this subgroup will commence specific motor control training in non-weight bearing positions which will then be progressed into functional exercises to be completed at home.

For participants in the zygapophyseal joint dysfunction subgroup, the focus of the specific physiotherapy treatment will be manual therapy [45]. Available techniques will include passive accessory movements, passive physiological rotation mobilisation, and high velocity thrust rotary manipulation [45]. Detailed clinical reasoning processes will be facilitated via the clinical notes to assist physiotherapists to select the most appropriate manual therapy techniques, and then to progress them appropriately based on the participant's clinical response [45]. Physiotherapists will have flexibility to explore other manual treatment techniques based on the results of a "mini-treatment", however the utilisation of key clinical reasoning principles will be mandatory. As per the reducible discogenic pain subgroup, specific motor control training will be integrated into a home based functional exercise program later in the treatment protocol.

The multi-factorial persistent pain subgroup will receive specific physiotherapy treatment based on an alternative treatment paradigm to the other pathoanatomical subgroups. Unlike the other subgroups, there will be minimal use of treatment strategies relating to pathoanatomical mechanisms. The focus of treatment for this subgroup will be i) detailed education in relation to the neurophysiology of pain and maladaptive central processing [60,61]; and b) a functional restoration program to promote graded exercise and increased function [62,63]. In addition, there will be an emphasis on the use of cognitive restructuring and behavioural strategies targeting key barriers identified via the Orebro Musculoskeletal Pain Questionnaire.

While the main focus of the specific physiotherapy treatment protocol will differ for each subgroup in the trial, several mandatory and optional treatment components will be common across subgroups. These include education regarding treatment options and expected recovery timeframes, goal setting, pacing, graded activity, cognitive restructuring and behavioural modification strategies, pain management strategies, sleep management, relaxation strategies, and management of inflammation (see Table 2).

### Participating physiotherapists

Physiotherapists from within a network of private practices (Spinal Management Clinics of Victoria) will provide the treatment for both groups. To be eligible, physiotherapists will need to have worked within the network for at least six months, have completed an initial two-day training program and be engaged with an ongoing clinical mentoring program.

All physiotherapists will undergo an additional one-day training program led by one of the researchers (JF) where the assessment and treatment protocols will be taught. This will be supplemented by an extensive manual outlining all assessment, treatment and trial protocols. For the duration of the trial a monthly teleconference will be undertaken for 60 minutes involving all treating physiotherapists to review specific cases in the context of the treatment protocols. Evaluation of treatment integrity and compliance by the physiotherapists for both the advice and the specific physiotherapy interventions will be achieved by checking the physiotherapist's clinical notes for each participant at week four, week seven and week ten of their program.

### Outcome assessment

Outcomes will all be assessed via a booklet of self-administered questionnaires that will be mailed to participants prior to the initial physical examination and at each of the follow-up points (5 weeks, 10 weeks, 26 weeks and 52 weeks post randomisation). Participants will return completed follow-up questionnaires to the researchers via mail marked only with their participant identification number. The outcomes to be measured in the trial are summarised in Table 3.

**Table 3:** Outcome measures

Outcome measure	Measurement point (weeks)
**Primary outcome measures**	
1. Oswestry Disability Index V2.1 with "sex life" question replaced by a "work/housework" question	0, 5, 10, 26, 52
2. Numerical rating scale for back pain (0-10)	0, 5, 10, 26, 52
3. Numerical rating scale for leg pain (0-10)	0, 5, 10, 26, 52
**Secondary outcome measures**	
1. Global rating of change scale (7-point Likert scale)	5, 10, 26, 52
2. Satisfaction with physiotherapy treatment (5-point Likert scale)	5, 10, 26, 52
3. Satisfaction with *results *of physiotherapy treatment (5-point Likert scale)	5, 10, 26, 52
4. Number of work days missed in the last 30 days	0, 5, 10, 26, 52
5. Interference with work or housework in the past week (5-point Likert scale)	0, 5, 10, 26, 52
6. Quality of life (EuroQol-5D)	0, 5, 10, 26, 52
7. Orebro musculoskeletal pain questionnaire	0, 5, 10, 26, 52
8. Sciatica frequency scale	0, 5, 10, 26, 52
9. Sciatica bothersomeness scale	0, 5, 10, 26, 52

#### Primary outcome measures

Activity limitation will be evaluated using nine questions from the Oswestry Disability Index version 2.1 [64], with the tenth question relating to "sex life" being replaced by a question relating to "work/housework" [65,66]. Rasch analysis has shown that this modified version performs as well as the original Oswestry [65], but it is aimed at preventing missing responses to the "sex life" question. The Oswestry has been shown to be a reliable, valid and responsive instrument for measuring activity limitation in people with low back pain and referred leg pain [67-69].

Separate 0-10 numerical rating scales (NRS) will be used to measure the average intensity of back pain and leg pain over the past week, with end-point descriptors of "no pain" and "worst pain possible" [70,71]. The NRS has good reliability [72,73], responsiveness [74,75] and validity [76,77].

#### Secondary outcome measures

Global rating of change will be measured using a 7-point Likert scale, with participants rating their overall change since the baseline assessment as "completely recovered", "much improved", "slightly improved", "no change", "slightly worsened", "much worsened", or "vastly worsened" [78,79]. Various versions of this scale are considered to be reliable, responsive and valid [79,80].

In addition to global rating of change, participants will rate their satisfaction with physiotherapy treatment and their satisfaction with the results of physiotherapy treatment on separate 5-point Likert scales, with ratings from "very satisfied" to "very dissatisfied" [81-83]. These scales have good reliability, validity and responsiveness [84,85].

The Sciatica Frequency and Bothersomeness Scale will be used to assess the frequency and "bothersomeness" of a range of leg symptoms including leg pain, numbness or tingling, and weakness in the leg or foot [86]. This scale has been used in a number of trials, particularly those focussing on sciatica or disc herniation [56,87-89]. The scale is considered to be a specific quality of life measure and has compared favourably to the generic SF-36 in people with LBD [86]. It has been shown to be reliable [86,90], responsive, and valid [90,91].

Interference with work due to the LBD will be assessed in two ways. Firstly, at each assessment point participants will record the number of work days missed due to their back/leg condition over the previous 30 days [81,82]. Secondly, participants will rate the degree of interference with work (employment or housework) caused by their back/leg condition over the previous week on a five point scale ranging from "not at all" to "extremely" [81,82]. These measurement methods have demonstrated good reliability, validity and responsiveness [84,85].

The Orebro Musculoskeletal Pain Questionnaire will be used as a measure of psychosocial risk factors [92,93]. Although it is more commonly used as a prognostic screening tool at one point in time [94], in our trial the Orebro will also be administered at each follow-up point to detect changes in psychosocial risk factors over time. This appears justified given that the Orebro has good test-retest reliability and internal consistency [92,95], although its responsiveness as an outcome measure is unclear.

Health-related quality of life will be measured with the EuoQol-5D [96]. Utilities will be calculated according to the validated algorithms of Dolan [97]. The EuroQol-5D has good reliability, validity and responsiveness [98-100], is a recommended outcome measure for low back pain research [82] and has been used in several other low back pain trials [101-103].

### Adverse events

Adverse events will be measured in both groups using two methods. Firstly, physiotherapists will record any adverse events that occur during the treatment period on their standardised clinical notes for each participant, and these will be submitted to the researchers after four weeks, seven weeks and at the conclusion of the intervention period (ten weeks). In addition, an open question on all follow-up questionnaires will ask participants to describe any adverse, harmful or unpleasant effects that they attribute to the intervention.

### Participant compliance and co-interventions

The number of physiotherapy treatment sessions attended by each participant, and the number of missed or cancelled appointments, will be recorded by the treating physiotherapists. Participant compliance with their physiotherapist's advice and prescribed exercises will also be reviewed by physiotherapists at each visit via direct questioning and reviewing participants' exercise charts. Information regarding the nature and degree of co-interventions as well as medication usage will be obtained from participants on each follow-up outcome questionnaire.

### Data integrity

All questionnaire data will be scored and entered into a computer spreadsheet by a researcher blinded to the group allocation of the participant. Data will be checked for omissions and outliers to identify potential data entry errors and these will be clarified with the data enterer.

### Blinding

Given the nature of the interventions it will not be possible to blind participants or physiotherapists. However, all physiotherapists and participants will be informed that both treatment approaches are valid interventions that have a realistic chance of being beneficial and that neither approach is known to be more effective than the other. Physiotherapists will also be instructed to treat participants in both groups with the same degree of rigor, enthusiasm and optimism.

### Data analysis

Data analysis will focus on detecting the between-group treatment effect (with 95% confidence intervals) at each of the follow-up points (5 weeks, 10 weeks, 26 weeks and 52 weeks following randomisation). Analyses will be conducted using PASW Version 18^b^, with alpha set at 0.05 using a two-tailed hypothesis. Continuous data will be analysed using linear mixed models (with the group x time interaction estimating the treatment effect). These were chosen for their strength in analysing longitudinal biological data and accounting for correlations associated with repeated measurements [104-106]. The mixed models will adjust for the baseline score of the outcome of interest, along with the stratification variables (treatment centre and subgroup) as recommended by the revised CONSORT statement [107]. The inclusion of treatment centre as a random effect will account for the potential clustering of outcomes within treatment centres [108,109]. In addition, the mixed models will adjust for gender and Orebro Musculoskeletal Pain Questionnaire score, as gender and psychosocial factors are considered to be important prognostic factors [107]. Ordinal data will be analysed using the Mann Whitney U test.

At each follow-up point, participants in each group will be dichotomised according to whether they achieved the minimum clinically important difference of the outcome or not, and then the risk ratio, risk difference and number needed to treat will be calculated along with 95% confidence intervals [107,110]. Statistical significance will be evaluated using Chi square analysis. For these purposes, the minimum clinically important difference will be defined as 10/100 for the Oswestry [111], 2/10 for the NRS pain scales [111], at least "much improved" on the global rating of change scale [71,112] and "very satisfied" on the treatment satisfaction scales [71]. It has been argued that these values for minimum clinically important difference may be too low in some contexts [113], hence we will repeat this analysis using a threshold of 50% reduction in Oswestry scores and NRS pain scores based on empirical validation studies suggesting that this may be a more suitable threshold for important differences [67,114].

All data will be analysed on an intention to treat basis, in that all participants will be analysed in the treatment group to which they are initially allocated regardless of their compliance with that treatment [107,115]. All participants who withdraw from treatment for any reason will continue to be contacted for follow-up assessments and informed that their data are still required. Our primary method of analysis will not impute missing data [116]. This is justified as all methods of data imputation have limitations [107], and the linear mixed model analysis that we planned for analysing continuous data is thought to inherently account for missing data in a more effective and less biased manner than data imputation methods [105,106,117,118]. However, given the popularity of simple data imputation methods [105,107] we will undertake a secondary sensitivity analysis to determine whether the results would differ if missing data were replaced using the last observation carried forward method.

In addition to the analyses described above, subgroup analyses will be undertaken to estimate the effects of specific treatment on each of the five subgroups within the trial. This will be possible due to the stratification of the randomisation for subgroup which will ensure that each subgroup has a balanced allocation of participants between the specific treatment and advice groups. Preliminary analysis will need to be undertaken on two of the subgroups prior to the completion of the trial in order for PhD thesis submission deadlines to be met. It is often recommended that an alpha adjustment be made when interim analyses are to be performed [107,119]. However, in our trial the overall data from all trial participants will not be analysed prior to the completion of recruitment, so no adjustment in alpha will be made in the final analysis involving all participants.

### Sample size

We will recruit a total sample size of at least 250 participants. We would require 128 participants (64 in each group) to detect the minimum clinically important difference between groups of 10% on the Oswestry assuming a standard deviation of 20 (two tailed hypothesis, alpha = 0.05, power = 80%) [120]. However, we wish to recruit more than this to improve power in our planned subgroup analyses. We acknowledge that the subgroup analyses are likely to remain underpowered [121], but they may provide some guidance for future research targeting the subgroups with greatest feasibility and effectiveness.

## Discussion

In this randomised controlled trial we aim to compare specific physiotherapy treatment to advice in people with LBD classified into five subgroups. We hypothesise that participants who receive specific physiotherapy treatment according to their subgroup will achieve superior clinical outcomes to those who receive physiotherapy advice. We will be testing this hypothesis in participants with subacute, non-compensable LBD. This decision has been made to avoid inclusion of participants with negative prognostic indicators including chronic symptoms [6,122,123] and compensable injuries [124,125], as these participants may require more extensive intervention than either of the treatment arms offer in our trial. We will avoid including participants with acute symptoms as this may lead to difficulty in demonstrating treatment effects above the higher rate of initial spontaneous recovery that might be expected in these participants [4,6,7,27]. Importantly, there has been limited research into the management of subacute LBD [27], and developing effective treatments for this group represents an opportunity to prevent the transition to chronic symptoms [126].

We will be utilising several strategies to maximise and assess treatment integrity for both the specific physiotherapy treatment group and the advice group [127,128]. A comprehensive treatment manual, initial training of physiotherapists, a monthly teleconference involving all treating physiotherapists, clinical notes directing physiotherapists along decision making algorithms, reviewing the clinical notes of every participant at three points during their treatment program, and the use of standardised participant information sheets are methods chosen to ensure that all participants receive treatment from physiotherapists that is standardised, accountable, and reproducible [127,128]. However, the algorithmic approach permits some flexibility in the selection and implementation of treatment components to ensure that treatment is relevant and specific for as many participants as possible. One example of this flexibility lies with exercises that can be implemented as part of the specific motor control training inherent in the protocols for all subgroups. Once improved motor control has been achieved in non-weight bearing positions, physiotherapists will be encouraged to design exercises relevant to each participant's functional goals. A participant with an interest in returning to golf for example could be taught a progression of resisted trunk rotation exercises whilst activating the transversus abdominis. This could progress to putting with a golf club, followed by chipping, followed by attending the driving range and finally progressing to the golf course.

The absence of a placebo control, along with the different number of sessions that will be provided to each group, could be perceived as limitations of the trial. However, this will be a pragmatic RCT comparing our classification-based treatment protocols to advice in its usual clinical form. Advice is typically administered over 1-2 sessions [35-37], and in this form it is known to be effective [35-37] and recommended in all international LBD guidelines [34]. Other LBD trials that have involved a similar imbalance in the number of sessions delivered to each group have found no differences in outcomes [129-131]. It has also been shown that the placebo effect (in comparison to no treatment) typically accounts for only small standardised mean differences of approximately 0.3 for participant-reported pain outcomes, equivalent to 3.2-6.5 points on a 100 point pain scale [132-134].

Given the nature of the interventions, it is not possible to blind physiotherapists and participants in this trial, although blinded scoring and entry of outcome questionnaires will be employed. We will however educate physiotherapists and participants regarding the validity of both treatment arms and inform them that both have a realistic chance of benefiting participants. We will also inform them that there is no existing evidence to suggest that one treatment approach is superior to the other.

We know of one completed trial [135] and two trials currently in progress [136,137] using classification principles to direct the treatment approach for participants. While the principle of sub-grouping is being utilised in all of these trials, each targets different subgroups, different treatment protocols and different populations to ours. The results of these trials will be of interest in determining which classification and treatment protocols have the greatest potential to benefit people with LBD.

We hope to complete enrolment for the trial by the end of 2011, with all 12-month follow-up data expected by the end of 2012.

## Competing interests

This is an investigator initiated trial. Funding for advertising and administration of the trial has been obtained via internal La Trobe University Grants. Spinal Management Clinics of Victoria (a division of LifeCare Health) and the treating physiotherapists will provide treatment free of charge for all trial participants. AH, LS, AC, MR, ST are consulting physiotherapists for Spinal Management Clinics of Victoria, and are also higher degree research students at La Trobe University. JF is a director of Spinal Management Clinics of Victoria, and director of the Low Back Pain Research Team at La Trobe University. RH and NT have no association with Spinal Management Clinics of Victoria. All investigators will maintain full autonomy and involvement in the design, conduct and reporting of the trial, with all having full access to the final data.

## Authors' contributions

JF, AH, LS, MR, AC, ST and RH were involved in the conception of the study. AH, JF and RH designed the trial protocol. JF, AH, LS, MR, AC and ST secured funding. JF developed the assessment and treatment protocols and trained the trial physiotherapists, with contributions from AH, LS, MR, AC and ST. AH and NT developed the statistical analysis strategy. NT prepared the randomization schedule. AH drafted the manuscript and JF, LS, MR, AC, ST, RH and NT contributed to the manuscript. All authors read and approved the final manuscript.

## Pre-publication history

The pre-publication history for this paper can be accessed here:

http://www.biomedcentral.com/1471-2474/12/104/prepub
